# Maximizing Polysaccharides and Phycoerythrin in *Porphyridium purpureum* via the Addition of Exogenous Compounds: A Response-Surface-Methodology Approach

**DOI:** 10.3390/md22030138

**Published:** 2024-03-21

**Authors:** Sanjiong Yi, Ai-Hua Zhang, Jianke Huang, Ting Yao, Bo Feng, Xinghu Zhou, Yadong Hu, Mingxuan Pan

**Affiliations:** 1 Jiangsu Province Engineering Research Center for Marine Bio-Resources Sustainable Utilization, College of Oceanography, Hohai University, Nanjing 210098, China; 18136562630@163.com (S.Y.); 15850576061@163.com (T.Y.); hhu335338350@163.com (B.F.); 2 Jiangsu Innovation Center of Marine Bioresource, Jiangsu Coast Development Investment Co., Ltd., Jiangsu Coast Development Group Co., Ltd., Nanjing 210019, China; zhouxinghu@jsyhkf.com (X.Z.); panmingxuan@jsyhkf.com (M.P.)

**Keywords:** *Porphyridium purpureum*, polysaccharide, phycoerythrin, response surface methodology, polypeptides, gluconate

## Abstract

Phycoerythrin and polysaccharides have significant commercial value in medicine, cosmetics, and food industries due to their excellent bioactive functions. To maximize the production of biomass, phycoerythrin, and polysaccharides in *Porphyridium purpureum*, culture media were supplemented with calcium gluconate (CG), magnesium gluconate (MG) and polypeptides (BT), and their optimal amounts were determined using the response surface methodology (RSM) based on three single-factor experiments. The optimal concentrations of CG, MG, and BT were determined to be 4, 12, and 2 g L^−1^, respectively. The RSM-based models indicated that biomass and phycoerythrin production were significantly affected only by MG and BT, respectively. However, polysaccharide production was significantly affected by the interactions between CG and BT and those between MG and BT, with no significant effect from BT alone. Using the optimized culture conditions, the maximum biomass (5.97 g L^−1^), phycoerythrin (102.95 mg L^−1^), and polysaccharide (1.42 g L^−1^) concentrations met and even surpassed the model-predicted maximums. After optimization, biomass, phycoerythrin, and polysaccharides concentrations increased by 132.3%, 27.97%, and 136.67%, respectively, compared to the control. Overall, this study establishes a strong foundation for the highly efficient production of phycoerythrin and polysaccharides using *P. purpureum*.

## 1. Introduction

*Porphyridium purpureum*, a member of the Rhodophyta, has attracted significant attention as a source of high-value bioactive substances, such as phycobiliproteins [1,2], long-chain polyunsaturated fatty acids [3,4], and sulfated polysaccharides [5]. Phycobiliproteins are crucial components of light-harvesting pigments in Cyanobacteria, Rhodophyta, and Cryptophyta [6]. According to their spectroscopic properties, phycobiliproteins with a pink/red coloration are classified as phycoerythrin (PE, 540–570 nm); those with blue coloration are phycocyanin (PC, 610–620 nm); and those with bluish-green coloration are allophycocyanin (APC, 650–655 nm) [1]. B-phycoerythrin and R-phycocyanin are present in *P. purpureum* [7]. Studies have demonstrated that phycoerythrin has significant antioxidant [8], immune-regulating [9], and anticancer effects [10]. Phycoerythrin extracted from *P. purpureum* is predominantly used in the food industry and has been approved as a food colorant due to its health benefits, intense fluorescence, and vivid color [11]. Furthermore, the polysaccharides derived from *P. purpureum* have seen a wide range of applications in the medical, cosmetics, and food industries [12,13] due to their excellent anti-inflammatory, anti-viral, anti-oxidant, and immunomodulating properties [14,15,16]. The commercial price of phycoerythrin varies significantly depending on its purity, but the market price of purified phycoerythrin was most recently reported to be USD 200 per milligram [17]. Moreover, there has been a continuous increase in the market demand for phycoerythrin in recent years owing to it being a natural product and having various functional properties [18]. However, the instability of phycoerythrin under adverse conditions such as high temperatures, low pH, and exposure to light remains a critical issue that limits its widespread application [19].

To date, much research has been conducted regarding how to enhance the concentrations of phycoerythrin and polysaccharides in *P. purpureum* to better meet market demands. The main strategies have focused on improving the culture media [1], supplying exogenous substances [20], optimizing environmental factors [21], and changing the culture method [22]. The optimization of the culture medium is a primary method for enhancing microalgal growth and increasing the production of bioactive substances. Nitrogen is a crucial nutrient for microalgal growth, and both nitrogen concentration and nitrogen type can impact the production of biomass and bioactive substances in microalgae [23]. This connection was exemplified by the work of Sánchez-Saavedra et al. [24], who found that the biomass productivity (173.2 mg L^−1^ d^−1^) of *P. cruentum* was higher when the alga was cultured at a NaNO_3_ concentration of 0.075 g L^−1^ compared to higher NaNO_3_ concentrations (i.e., 0.45 and 0.225 g L^−1^). Additionally, the presence of an organic carbon source can influence the yields of biomass and bioactive substances in *P. purpureum* [23]. For instance, the maximum biomass of *P. purpureum* CoE1 was achieved with a 0.5% (*w*/*v*) glucose dosage, while the maximum arachidonic acid (ARA) concentration was obtained with a 0.38% (*w*/*v*) glycerol dosage [22]. Furthermore, adding exogenous substances is essential for maximizing microalgal biomass and the concentrations of bioactive substances. Numerous studies have investigated how the biomass and the production of bioactive substances are enhanced by supplementing with phytohormones, metal ions, and vitamins [25,26,27]. One such study showed that the ARA concentration of *P. purpureum* was enhanced by stimulation with 20 mg L^−1^ of 5-aminolevulinic acid, with a peak yield of 170.32 mg L^−1^, which represented a 70.82% increase compared to the control [20]. Hence, it is essential to add the suitable substances to culture media at concentrations within the appropriate ranges to maximize the production efficiency of active substances.

Response surface methodology (RSM) is an important statistical optimization tool that has been widely used for experimental modeling. This method reduces the number of experiments required and optimizes the interactions among the experimental process parameters in various processes [28]. Previously, RSM has been utilized to optimize the microalgae-culture process and significantly improve microalgal productivity. The optimal concentrations of sodium chloride, magnesium sulfate, sodium nitrate, and dipotassium hydrogen phosphate have been determined using the RSM, with the highest PE content in *P. purpureum* reaching 3.3% under optimized conditions [1]. To maximize the PB content of *P. cruentum*, RSM was used to determine the optimal conditions of temperature (10 °C) and light intensity (30 μmol m^−2^ s^−1^), resulting in a maximum phycobiliprotein (PB) content of 2.9% [29]. Clearly, RSM has been proven to be an efficient and effective method for medium optimization.

A commercially produced combination of peptide complexes, commonly referred to as Bainengtai (BT) in China, is composed of enzymatic hydrolysates of high-quality plant proteins. This product is extensively used in the agriculture and feed industries to promote the growth of both plants and animals. Our previous study showed that BT enhanced phycocyanin production in *Arthrospira maxima* [30]. Calcium gluconate (CG) and magnesium gluconate (MG) dissociate into gluconic acid and cations in the medium solutions, so they can be considered to be a combination of a glucose and an ion under appropriate conditions [31]. Similarly, gluconate is primarily used as an additive in the food, pharmaceutical, health, and construction industries. Pang et al. [32] indicated that gluconate, the metabolic product of glucose, significantly increased the biomass of *Haematococcus pluvialis* compared to sodium acetate and ribose, making it a suitable candidate for use as an organic carbon source.

Therefore, in the present study, we investigated the effects of calcium gluconate (CG), magnesium gluconate (MG), and BT as additional supplements in *P. purpureum* culture. We aimed to determine the independent and interactive effects of the three factors (CG, MG, and BT concentrations) on biomass, phycoerythrin, and polysaccharide production by *P. purpureum*. Additionally, using RSM, this study focused on determining the optimal amounts of these substances to maximize biomass yield and the production of phycoerythrin and polysaccharides.

## 2. Results

### 2.1. Effects of Single Factors (CG, MG, and BT) on Microalgal Growth and the Accumulation of Bioactive Substances 

**Figure 1:**
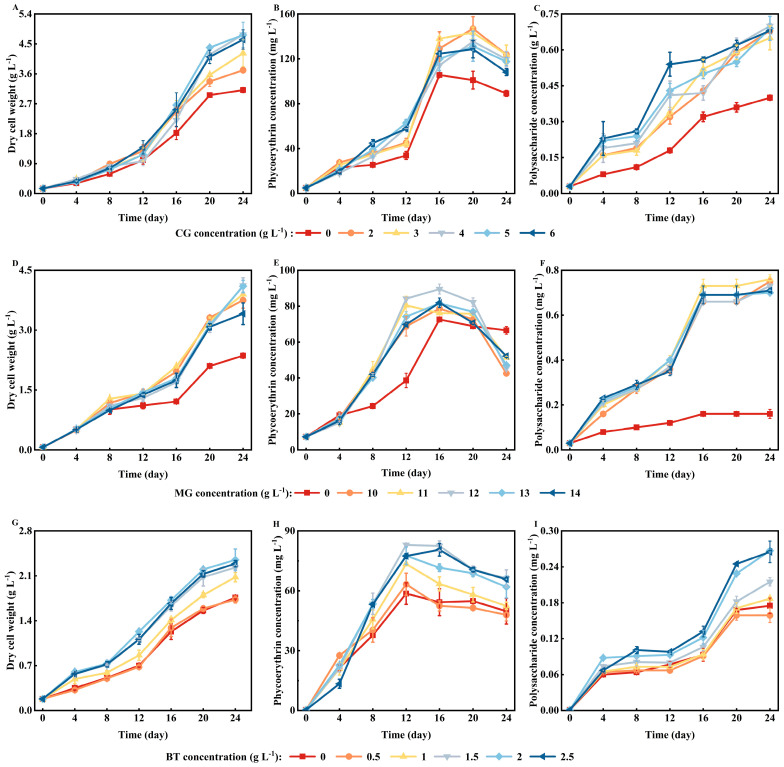
Effects of CG (**A**–**C**), MG (**D**–**F**), and BT (**G**–**I**) on the production of biomass, phycoerythrin and polysaccharides by *P. purpureum*. The data represent the average ± standard deviation (*n* = 3). CG, calcium gluconate; MG, magnesium gluconate; BT, polypeptides.

It was found that the CG supplementation promotes microalgal growth and the accumulation of bioactive substances. As shown in Figure 1A,C, among all CG concentration levels, microalgal biomass and polysaccharide concentrations peaked when CG was added at 4 g L^−1^. After 24 days of culturing, the maximum concentrations of biomass and polysaccharides reached 4.78 ± 0.03 and 0.7 ± 0.01 g L^−1^, respectively, which were 1.54 and 1.75 times higher than the concentrations in the control group. However, the maximum phycoerythrin concentration, which was 1.45 times higher than that in the control group (Figure 1B; 146.9 ± 10.77 mg L^−1^, day 20), was observed with a CG concentration of 2 g L^−1^. These results indicated that the optimal CG concentration for *P. purpureum* growth and polysaccharide accumulation was 4 g L^−1^, whereas a CG concentration of 2 g L^−1^ was optimal for phycoerythrin production.

MG supplementation also enhanced the production of biomass and bioactive substances in *P. purpureum*. As shown in Figure 1F, the addition of MG markedly increased the polysaccharide yield, which reached 0.76 ± 0.02 g L^−1^ on the 24th day. Compared to the control group, the maximum concentration of polysaccharide increased by 375% at an MG concentration of 11 g L^−1^. In addition, the biomass and phycoerythrin concentrations (Figure 1D,E) peaked at 4.12 ± 0.19 g L^−1^ and 89.53 ± 2.77 mg L^−1^ on days 24 and 16, respectively. Compared to the control group, the maximum biomass and phycoerythrin concentrations increased by 74.6% and 23.3%, respectively, at an MG concentration of 12 g L^−1^. As a consequence, an MG concentration of 12 g L^−1^ was identified as the optimal concentration of MG for *P. purpureum* growth and phycoerythrin accumulation. However, for polysaccharide production, an MG concentration of 11 g L^−1^ was optimal.

In terms of the impact of BT on microalgal growth and the accumulation of bioactive substances, the maximum biomass and phycoerythrin and polysaccharide concentrations in the BT treatment groups were generally higher than those in the control group, except at a BT concentration of 0.5 g L^−1^. On the 24th day, the biomass and polysaccharide concentrations reached their maximum values of 2.35 ± 0.17 g L^−1^ and 0.267 ± 0.002 g L^−1^, respectively, at a BT concentration of 2 g L^−1^ (Figure 1G,I). However, when the BT concentration was 1.5 g L^−1^, the concentration of phycoerythrin (Figure 1H) reached its maximum (83.02 ± 0.59 mg L^−1^) on the 12th day. Therefore, the optimal BT concentrations for *P. purpureum* growth and polysaccharide accumulation were both 2 g L^−1^, while that for phycoerythrin production was 1.5 g L^−1^.

### 2.2. Model Fitting of RSM

The quadratic regression equations for the biomass, phycoerythrin, and polysaccharide concentrations were obtained using RSM (Table 1). The *p*-values for all the investigated responses were *p* < 0.05, showing the significance of the applied model [33]. The *p*-values for biomass concentration (0.0123), phycoerythrin concentration (0.0054) and polysaccharide concentration (0.0054) were all less than 0.05, demonstrating that the models for all responses were significant. At the same time, the high R^2^ value (>0.8893) suggested that all the models fit the data well (Table 1). Furthermore, the relatively low variation coefficients (9.93–11.07%) and the lack of fit (*p* > 0.05) implied high experimental reliability and a strong correlation between the responses and the independent variables.

### 2.3. Combined Effects of Variables on Biomass, Phycoerythrin, and Polysaccharide Concentrations

**Figure 2:**
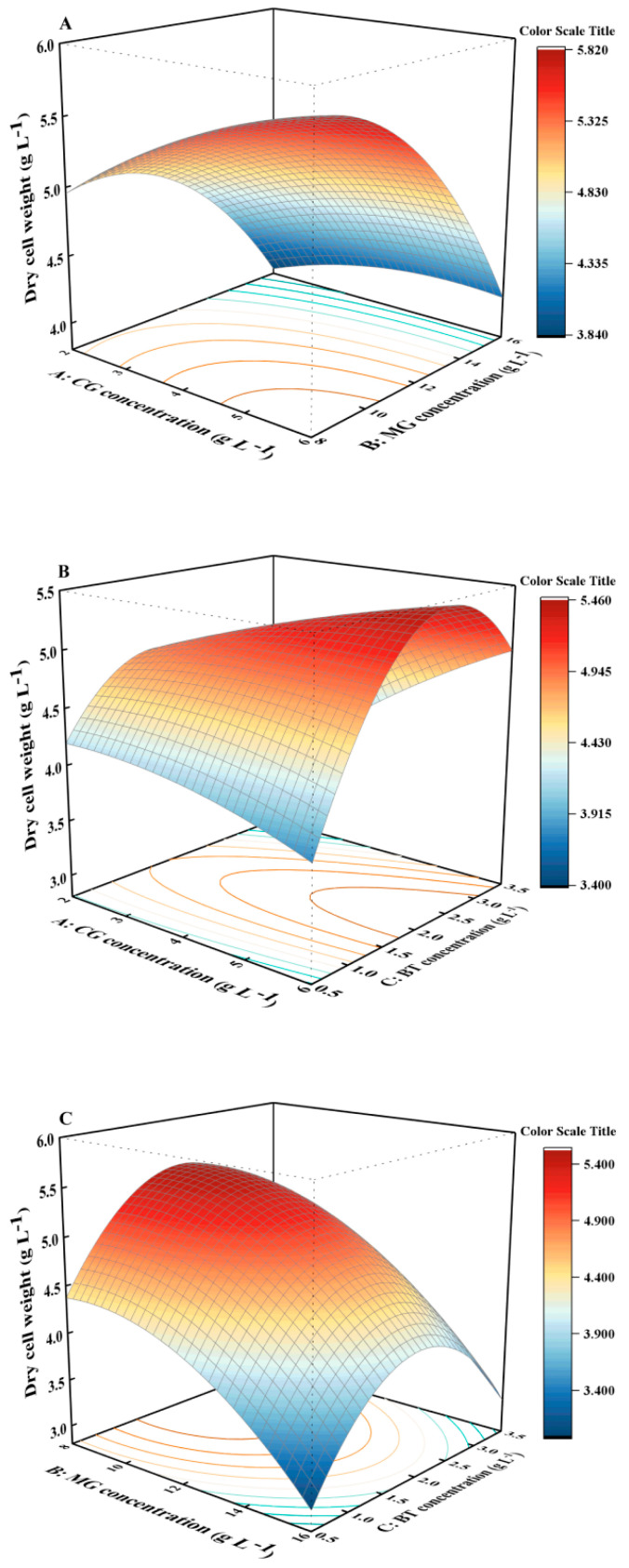
Response-surface plots showing the combined effect of the CG, MG, and BT concentration for responses in terms of biomass concentration. (**A**): interaction between CG and MG; (**B**): interaction between CG and BT; (**C**): interaction between MG and BT; CG, calcium gluconate; MG, magnesium gluconate; BT, polypeptide.

**Figure 3:**
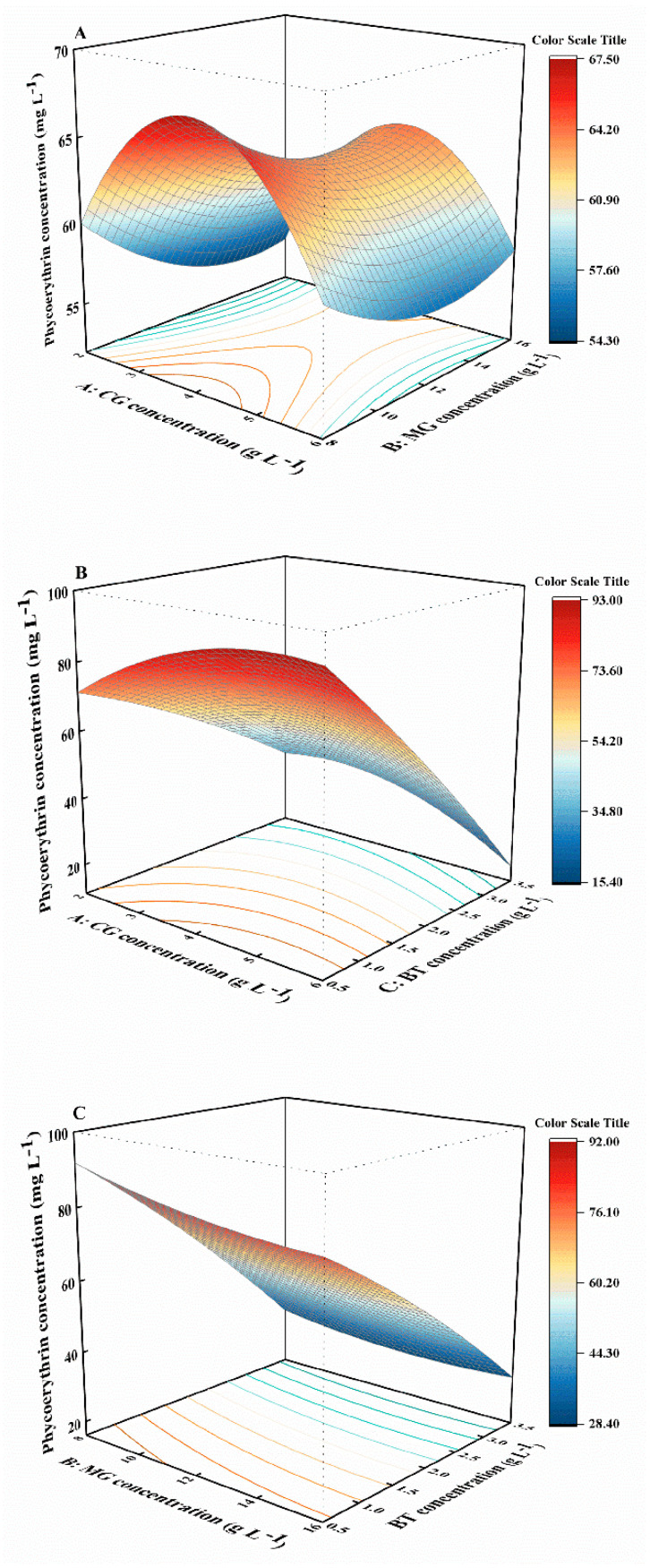
Response-surface plots showing the combined effect of the CG, MG, and BT concentration for responses in terms of phycoerythrin concentration. (**A**): interaction between CG and MG; (**B**): interaction between CG and BT; (**C**): interaction between MG and BT; CG, calcium gluconate; MG, magnesium gluconate; BT, polypeptide.

**Figure 4:**
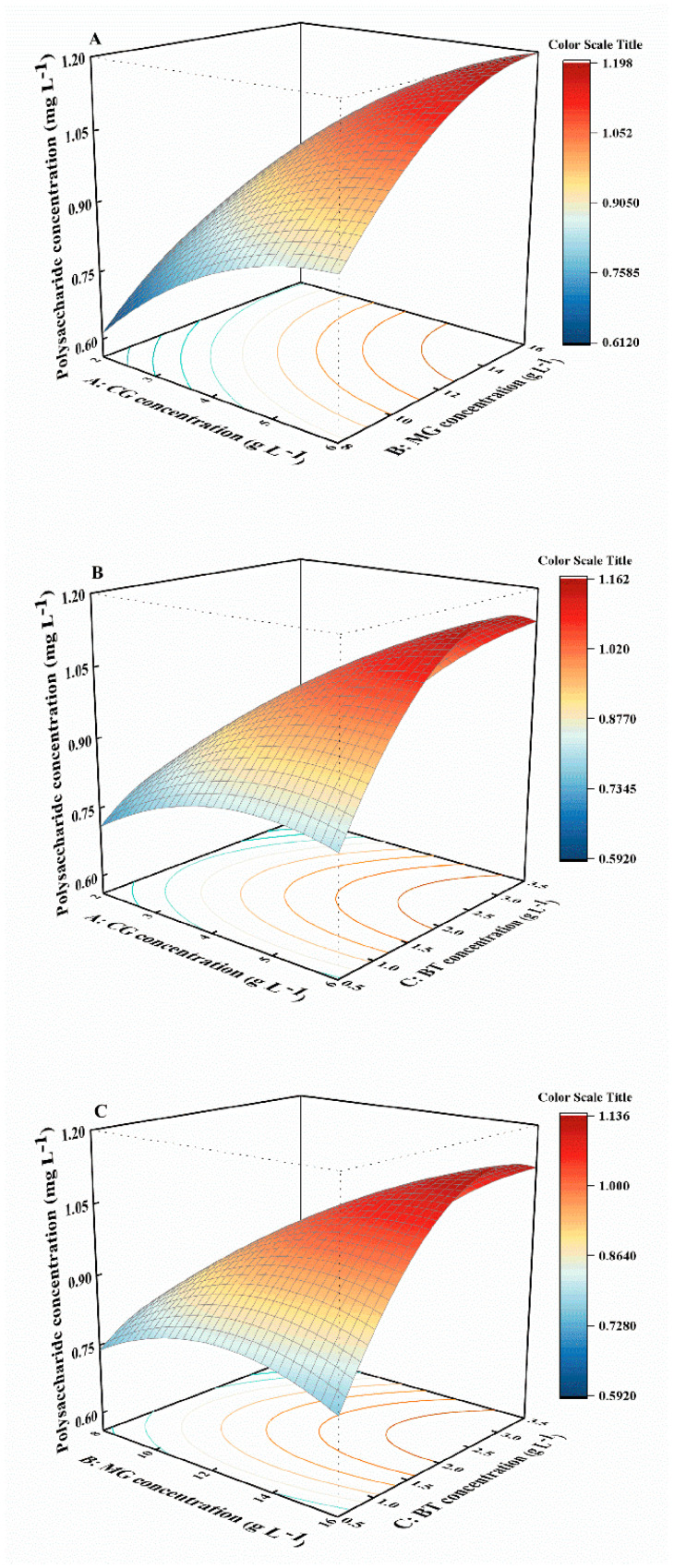
Response-surface plots showing the combined effect of the CG, MG, and BT concentration for responses of polysaccharide concentration. (**A**): interaction between CG and MG; (**B**): interaction between CG and BT; (**C**): interaction between MG and BT; CG, calcium gluconate; MG, magnesium gluconate; BT, polypeptide.

The biomass, phycoerythrin, and polysaccharide concentrations under various experimental conditions are shown in Table 2, and the relationships between the three variables and the responses are depicted in 3D response surfaces and contour plots in Figure 2, Figure 3 and Figure 4. The biomass concentration increased initially and then decreased with increasing BT concentration when the concentrations of CG and MG were fixed at 6 and 16 g L^−1^, respectively (Figure 2B,C). Higher biomasses were observed at lower MG concentrations when CG and BT were fixed at 6 CG and 0.5 g L^−1^, respectively (Figure 2A,C). In addition, the ANOVA results of the model indicated that single factors (A, C), interaction terms (AB, AC, BC), and quadratic terms (A^2^, B^2^) had non-significant effects on biomass concentration, with *p*-values of these factors all exceeding 0.05. The microalgal biomass was more sensitive to MG than to CG and BT (Table S1). In general, a lower MG concentration (8 g L^−1^) contributed to increased biomass production.

The relationships between the phycoerythrin concentration and the three independent variables were analyzed. The concentration of phycoerythrin declined with increasing BT concentration when the concentrations of CG and MG were fixed at 6 and 16 g L^−1^, respectively (Figure 3B,C). Meanwhile, the concentration of phycoerythrin first increased and then decreased with increasing MG concentration, but this response was dependent on CG concentration (Figure 3A). However, the ANOVA results for the model indicated that the single factors (A, B), interaction terms (AB, AC, BC), and quadratic terms (A^2^, B^2^) had non-significant effects on phycoerythrin production (*p* > 0.05). Compared to CG and MG, BT had a stronger influence on phycoerythrin production (Table S2). Therefore, the BT concentration range 1.5–2.5 g L^−1^ was identified as optimal for phycoerythrin accumulation.

In terms of polysaccharide concentrations, an initially increasing and then decreasing trend was observed as the BT concentration increased from 1.0 to 3.5 g L^−1^ when CG and MG concentrations were fixed at 6 and 16 g L^−1^, respectively (Figure 4B,C). The highest polysaccharide concentration occurred over the range of BT concentrations from 2.0 to 3.5 g L^−1^. However, polysaccharide concentration was less sensitive to BT (*p* > 0.05) than to CG and MG (*p* < 0.05). In addition, the ANOVA results of the model revealed that single factors (A, B), interaction terms (AB, AC), and quadratic terms (C^2^) had significant effects on polysaccharide concentration (*p* < 0.05) (Table S3). A strong interactive effect was observed between the concentrations of CG and BT, as well as between the concentrations of MG and BT, with significant *p*-values of 0.0472 and 0.0293, respectively. Overall, when the concentrations of CG and MG were held constant, relatively high BT concentrations (2.0–3.5 g L^−1^) were found to be more conducive to polysaccharide accumulation.

### 2.4. Optimization and Experimental Validation

**Figure 5:**
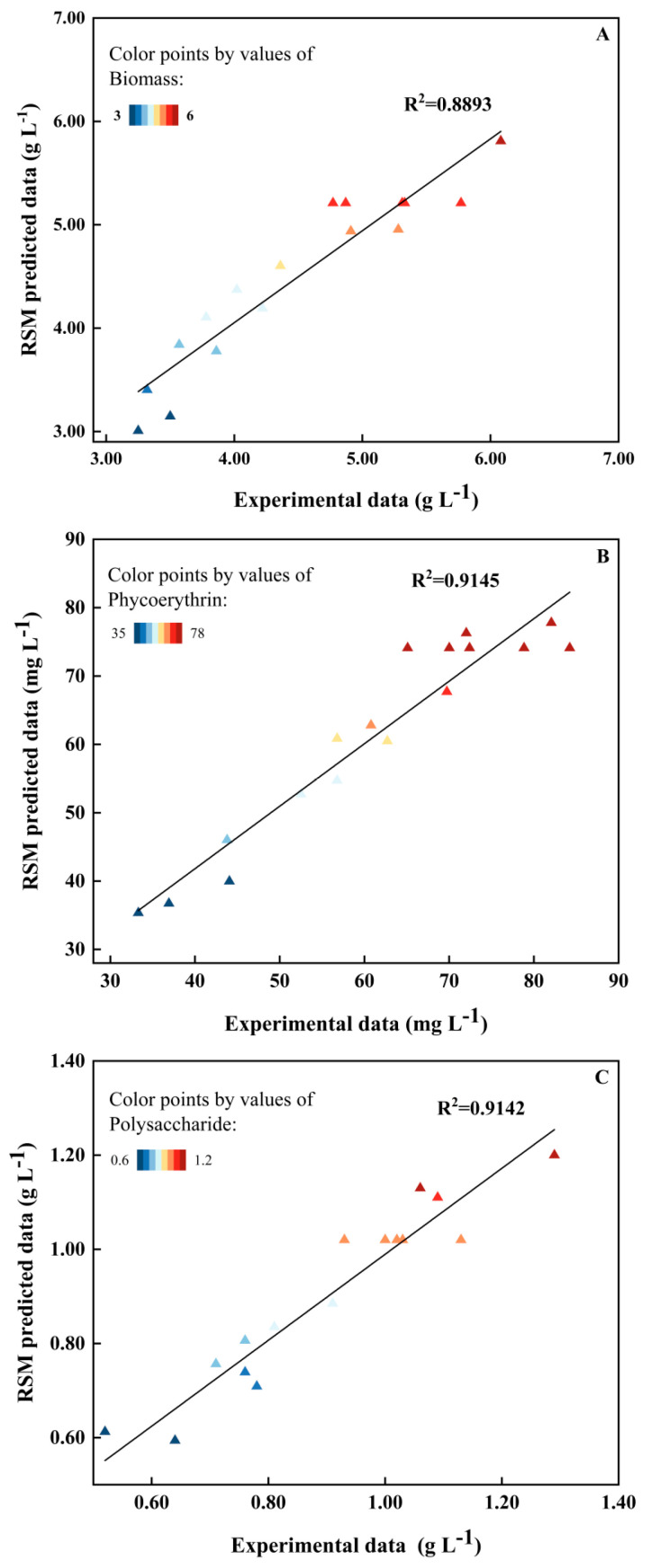
Comparison between experimental and predicted values of (**A**) biomass concentration, (**B**) phycoerythrin concentration, and (**C**) polysaccharide concentration.

**Figure 6:**
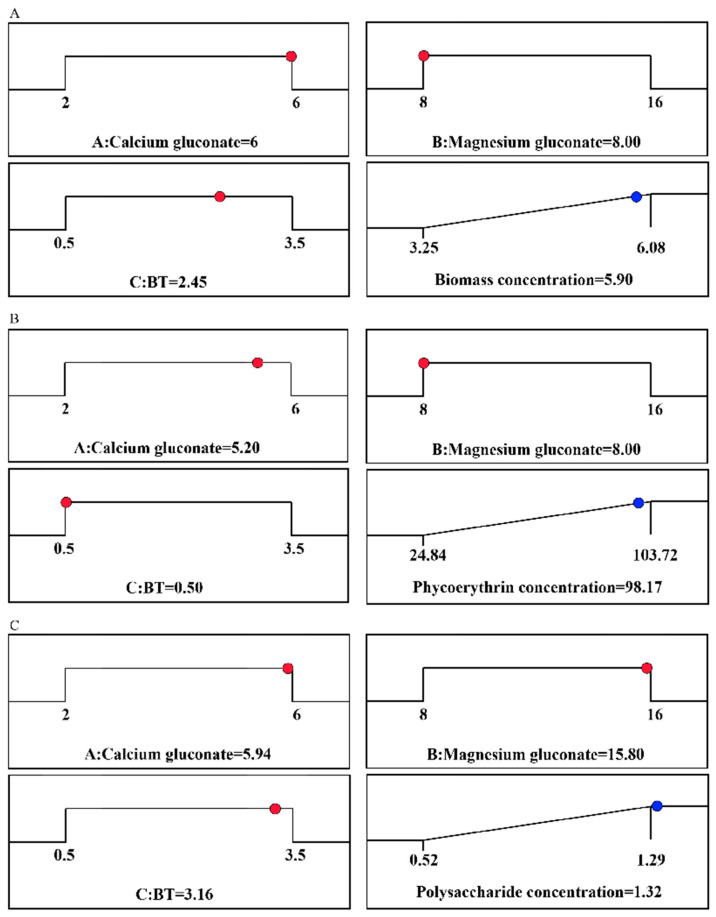
Optimal conditions predicted by the models for (**A**) biomass concentration, (**B**) phycoerythrin concentration, and (**C**) polysaccharide concentration. Red dot: the optimal addition amount predicted by the model; Blue dot: the optimal response concentration predicted by the model.

A comparison between the actual experimental data and the predicted data from the quadratic regression model is illustrated in Figure 5. The strong linear relationship between the two data sets indicated that the predictions aligned well with the experimental results, suggesting that the model is suitable for prediction and optimization. According to the model prediction, the maximum concentrations of biomass, phycoerythrin, and polysaccharides can reach as high as 5.90 g L^−1^, 98.17 mg L^−1^, and 1.32 g L^−1^, respectively, when the concentrations of CG, MG, and BT are at optimal levels (Figure 6). Indeed, these levels were achieved in the verification experiments, where the measured biomass, phycoerythrin, and polysaccharide concentrations all slightly exceeded their respective predicted concentrations, at 5.97 g L^−1^, 102.95 mg L^−1^, and 1.42 g L^−1^, respectively.

## 3. Discussion

In this study, the growth and accumulation of bioactive substances in *P. purpureum* were evaluated under supplementation with three exogenous substances (CG, MG, and BT). CG and MG dissociate into gluconic acid and cations in the medium solutions, so they can be considered a combination of a carbon resource (glucose) and an ion (calcium or magnesium) under appropriate conditions. The experimental results showed that the biomass and concentrations of phycoerythrin and polysaccharides increased with the addition of CG or MG compared to the control. This effect was attributed to the presence of gluconate, which acted as an organic carbon source. A similar trend was observed in *H. pluvialis*, where the addition of gluconate increased the biomass productivity and photosynthetic efficiency [32]. Furthermore, as for gluconate, the concentrations of calcium and magnesium can also affect biomass and the accumulation of bioactive substances. A study of *Chlorella vulgaris* and *Scenedesmus obliquus* found that an increasing magnesium concentration positively affected the biomass and lipid content [26]. In their study, compared to the control group, the biomass yield of *C. vulgaris* and *S. obliquus* increased by 33% and 36%, respectively, at 150 mg L^−1^ MgSO_4_. Furthermore, the lipid content increased to a maximum of 27% and 26% of dry cell weight in *C. vulgaris* and *S. obliquus*, respectively, at 100 mg L^−1^ MgSO_4_. However, the increased calcium concentrations had a little impact on the growth of the microalgae. Interestingly, the lipid content of *C. vulgaris* and *S. obliquus* peaked at 331 mg L^−1^ and 224 mg L^−1^, respectively, under calcium-starved conditions. Therefore, the combination of gluconate and metal ions can promote microalgal growth and the production of bioactive substances.

BT can be considered a nitrogen source and is primarily composed of polypeptides. Nitrogen is an essential nutrient for cell growth and is used to synthesize photosynthetic pigments, amino acids, coenzymes, and other compounds [24]. Therefore, the concentration of nitrogen can influence microalgal growth and the accumulation of bioactive substances. Over a gradient of NaNO_3_ in nitrogen-free Zarrouk medium, the highest cell density of *Arthrospira platensis* was observed at a concentration of 40 mM NaNO_3_, in the middle of the range [34]. Furthermore, the amounts of proteins and pigments in *A. platensis* decreased when the alga was cultured under conditions of nitrogen limitation [35]. In addition to nitrogen concentration, the type of nitrogen source can significantly affect the growth of microalgae and the accumulation of bioactive substances [36,37]. In the present study, as the BT concentration increased, the polysaccharides concentration gradually increased due to the increasing biomass concentration, but the change had little effect on the polysaccharide content (Table S4). However, after the initial increase, the biomass and phycoerythrin concentrations decreased as the BT concentration increased further, likely due to the high nitrogen concentration. Similar patterns were observed in *Neochloris oleoabundans*, whose growth was not enhanced at higher nitrate concentrations (15 and 20 mM) [38].

To date, most studies on *P. purpureum* have focused on the effects of individual exogenous substances and single or interactive environmental conditions on the production of biomass and high-value compounds [3,20,29]. To the best of our knowledge, this is the first study to investigate the interactive effects of exogenous substances on the growth of and accumulation of high-value compounds in *P. purpureum*. We evaluated the combined effects of exogenous substances on biomass, phycoerythrin production, and polysaccharide production in *P. purpureum* using the response surface methodology, applying a second-degree polynomial (i.e., quadratic model). This approach allowed us to determine the optimal supplement levels for maximizing the concentrations of biomass, phycoerythrin, and polysaccharides. MG was the only factor that exhibited a significant effect on biomass yield (*p* < 0.01). The MG concentration was negatively correlated with the biomass of *P. purpureum*, with the highest biomass occurring at a low MG concentration (8 g L^−1^). This result agreed with a previous report that the biomass of *P. purpureum* was 1.82 times higher at a glucose concentration of 5 g L^−1^ compared to a concentration of 10 g L^−1^ [22]. It was also reported that the biomass yields of *C. vulgaris* and *S. obliquus* peaked when the magnesium concentration increased to double that of the control [26]. BT was the only factor that had a significant effect on phycoerythrin production (*p* < 0.01). Similarly, the highest phycoerythrin concentration was achieved at an extremely low concentration of BT (0.5 g L^−1^). As a nitrogen source, BT enhanced the growth of microalgae and the accumulation of bioactive substances. In addition to influencing the growth of *P. purpureum*, the nitrogen source can also affect the synthesis of phycoerythrin. Nitrogen deficiency has been previously observed to decrease the content and stability of phycobilisomes associated with photosynthetic activity in *P. purpureum* [39]. It has also been reported that the maximum phycoerythrin concentration in *P. purpureum* UTEX LB 2757 occurred at a low nitrogen concentration of 0.075 g L^−1^ [17]. In this study, the production of polysaccharides was significantly affected by the interactions between CG and BT and between MG and BT (*p* < 0.05) but not by changes in BT alone (*p* > 0.05). The method of culture has also been observed to affect production, with increased biomass and production of bioactive substances by microalgae in mixotrophic culture compared to photoautotrophic and heterotrophic culture [40]. Moreover, there was an observed increase in oxidative phosphorylation and a weakening of photosynthesis in microalgal cells when an organic carbon source was added. Furthermore, the enhanced phosphorylation not only compensated for the loss of photosynthesis, but also substantially increased the biomass [41]. Compared to the microalgae in photoautotrophic culture, *Chlorella sorokiniana* showed increased biomass and production of bioactive substances due to changes in the metabolic genes involved, which were more closely related to carbon flux than to photosynthesis [42]. Therefore, it was speculated that the decreases in phycoerythrin and increases in polysaccharides induced by the organic carbon source were due to gene regulation in the related metabolic pathways.

In order to optimize the *P. purpureum* growth medium, we established predictor models for algal biomass, phycoerythrin, and polysaccharide concentrations using the response surface method. The maximum biomass (5.97 g L^−1^), phycoerythrin (102.95 mg L^−1^), and polysaccharide concentrations (1.42 g L^−1^) were successfully achieved using the ideal conditions predicted by this model. These values were 132.3%, 27.97%, and 140.33% higher than those achieved in the initial ASW medium, respectively.

To date, various studies have investigated approaches for improving biomass, phycoerythrin production, and polysaccharide production in *P. purpureum*; a summary of their results is provided in Table 3. As can be seen, there have been fewer studies on phycoerythrin production. The resulting polysaccharide concentrations varied greatly, ranging from 0.23 to 4.62 g L^−1^, with the majority concentrated in the range from 0.23 to 2.14 g L^−1^. The significant variation in polysaccharide concentration was likely caused by the diversity of culture conditions among studies, which differed in terms of algal strains, culture medium, light intensity, and other factors. As shown in Table 3, a glass flask and a bioreactor were the primary devices used for culturing *P. purpureum*. It is worth noting that higher polysaccharide production (>2 g L^−1^) was achieved in the small photobioreactors (<5 L) (Table 3) [21,43,44,45]. These high polysaccharide concentrations were attributed to differences in lighting conditions and were attained in photobioreactors rather than flasks. The polysaccharide concentrations in algae cultured in glass flasks (<1 g L^−1^) or relatively large-scale photobioreactors (<1.4 g L^−1^) were lower than that in our study (1.42 g L^−1^).

It should be noted that the addition of CG, MG, and BT will increase production costs. Therefore, a brief economic analysis was conducted as follows. Under optimal conditions, the phycoerythrin concentration reached the maximum levels, with a 22.5 mg L^−1^ increase in phycoerythrin yield compared to the control. The extra cost of the supplemental substances required for 1 L optimized culture medium was calculated based on the commercial prices of the substances (CG, MG, and BT) and came to ~USD 0.4 L^−1^. Considering the increased production of phycoerythrin (an additional 0.0225 g L^−1^) and the market price of purified phycoerythrin (USD 200 g^−1^), the extra production was valued at approximately USD 4.5 L^−1^, which far exceeded the additional input cost (USD 0.4). Therefore, this approach to maximizing phycoerythrin production would be cost-effective and economically feasible.

Overall, high productivity was achieved for both phycoerythrin and polysaccharide by adding CG, MG, and BT in quantities based on RSM to optimize the culture medium. However, the cultures in this study were limited to 500 mL flasks, so further testing would be required in scaled-up photobioreactors to confirm that these results are scalable. Therefore, the next step may involve using a larger-scale photobioreactor, investigating the influence of light intensity, and combining the photobioreactor with optimized light intensity to further maximize the production of phycoerythrin and polysaccharides.

## 4. Materials and Methods

### 4.1. Microalgal Strain

The marine microalgal strain *Porphyridium purpureum (Bory) K.M.Drew & R.Ross 1965* was obtained from the Freshwater Algae Culture Center at the Institute of Aquatic Biology (Wuhan, China) and was maintained in ASW medium [21] at 25 °C and a light intensity of 100 μmol m^−2^ s^−1^.

### 4.2. Experimental Design

#### 4.2.1. Experiments to Determine the Optimal Concentrations of Exogenous Substances

To investigate the effects of exogenous substances added to the initial culture media on biomass and the accumulation of bioactive substances, we conducted experiments with CG, MG, and BT. CG and MG were purchased from Shanghai Aladdin Biochemical Technology Company (Shanghai, China). and BT was purchased from Jiangsu Rishengchang Biotechnology Company (Nanjing, China). BT is a mixture of amino acids, polypeptides, and proteins; the detailed composition of BT has been reported previously [30].

For single-factor experiments, the six CG concentrations (0, 2, 3, 4, 5, and 6 g L^−1^), six MG concentrations (0, 10, 11, 12, 13, and 14 g L^−1^), and six BT concentrations (0, 0.5, 1, 1.5, 2, and 2.5 g L^−1^) were used. For the experiments, *P. purpureum* in the logarithmic growth phase were inoculated into 500 mL Erlenmeyer flasks containing 200 mL ASW medium. All culture media were pre-sterilized by autoclaving at 121 °C, 1 bar, for 20 min. The inoculation amount was 10% of the liquid volume load. After inoculation, the flasks were placed on a shaker at a speed of 170 rpm. *P. purpureum* was cultivated at 25 °C with continuous light (100 μmol m^−2^ s^−1^) for 24 days. The continuous light was provided by white LED lights (Philips Lighting, Shanghai, China), and the light intensity was measured by illuminometer. The biomass and the phycoerythrin and polysaccharide concentrations were measured every four days. Three parallel experimental replicates were established for all experimental treatment groups.

#### 4.2.2. Response Surface Experiments for Three Exogenous Substances

Based on the results of the single-factor experiments, a three-factor, three-level experiment was designed (Table 4). The single-factor experiments demonstrated that the addition of exogenous substances enhanced the biomass, phycoerythrin, and polysaccharide concentrations in *P. purpureum*. Therefore, the Box-Behnken Design (BBD) [50], a well-known statistical design for experiments, was chosen as the response surface method for optimization. The optimal concentrations of each factor, as determined by the single-factor experiments, were chosen as the central point of the BBD. The relationships between the dependent (biomass, phycoerythrin, and polysaccharide concentrations) and independent variables (A: CG, B: MG, and C: BT) were quantitatively determined. The experimental design and mathematical model were created using Design-Expert software (version 13.0.1.0), and the statistical analysis was conducted using the same platform.

According to the BBD, 17 sets of experiments were conducted (Table 2). Different concentrations of CG, MG, and BT were added to the ASW medium in each experimental group. The experimental conditions were the same as those detailed previously. It is worth noting that there were two parallel experimental replicates for each treatment setup in the response-surface experiments. validation experiments were conducted using the optimal conditions predicted by the RSM model with the aim of maximizing biomass, phycocyanin, and polysaccharide concentrations.

### 4.3. Dry Cell Weight

Dry cell weight (DCW) was determined using the dry-weight method [51]. In brief, a dry weighing disc was first weighed, with its weight represented as M_1_. Then, 5 mL of each microalgae solution (V) was harvested by centrifuging the cells at 8000 r min^−1^ for 5 min, washing them with deionized water once, centrifuging them again, then drying them in a 100 °C oven until they reached a constant weight. Then, the weighing disk was cooled and weighed, with its weight recorded as M_2_. Finally, the DCW was calculated as follows:

(1) $$DCW=\frac{M_{2}-M_{1}}{V}$$

where *DCW* is the biomass concentration (g L^−1^); *M*_2_ represents the weight of dried weighing disc with the sample (g); *M*_1_ represents the weight of pre-dried empty weighing disc (g); and *V* is the sampling volume (L).

### 4.4. Phycoerythrin Concentration

The concentration of phycoerythrin (PE) was determined spectrophotometrically. Firstly, a 5 mL sample of the *P. purpureum* culture was centrifuged at 8000 rpm for 5 min. After centrifugation, the supernatant was carefully drained off. Then, 5 mL of 0.1 mol L^−1^ phosphate buffer (pH 6.8) was added to resuspend the precipitated biomass. To break the microalgae cells and release the PE, the resuspended cells were subjected to three freeze-thaw cycles. Then, the mixture was centrifuged again at 5000 rpm for 5 min to collect the supernatant. The concentration of PE was determined by measuring the absorbance of the supernatant at 564 nm, 592 nm, and 455 nm. The concentration of phycoerythrin was determined using the following formula [52]:

(2) $$PE=[(OD_{564}-OD_{592})-(OD_{455}-OD_{592})\times 0.2]\times 0.12$$

where *PE* is the concentration of phycoerythrin in the microalgal solution (g L^−1^) and *OD*_564_, *OD*_592_, and *OD*_455_ are the absorbances at 564, 592, and 455 nm, respectively.

### 4.5. Polysaccharide Concentration

A 5 mL suspension of *P. purpureum* culture was subjected to three freeze-thaw cycles. The resulting extract solution was centrifuged at 5000 rpm for 5 min. The supernatant was collected so the polysaccharide concentration could be measured. The polysaccharide concentration of *P. purpureum* was determined using the sulfuric acid-phenol method [53]. The concentration of polysaccharides (Y, g L^−1^) was determined using a standard absorbance curve (R^2^ = 0.995).

(3) $$Y=\frac{A_{490}+0.0173}{7.1137}$$

### 4.6. Statistical Analysis

After all experiments had been conducted, the relationship between the dependent and independent variables was explained by the second-degree polynomial as shown by the following equation:

(4) $$y=\beta_{0}+\sum_{i=1}^{n}b_{i}x_{i}+\sum_{i=2}^{n}b_{ii}x_{i}^{2}+\sum_{j=i+1}^{n}b_{ij}x_{i}x_{j}$$

where *y* is the response; *β*_0_ is the intercept; *β_i_*, *β_ii_*, and *β_ij_* are the regression coefficients of different variables in linear and quadratic equations; *n* is the number of studied variables; and *x_i_* and *x_j_* represent independent variables.

Statistical differences were observed among the experiments, as determined by analysis of variance (ANOVA) and tests conducted in Design-Export software (version 13.0.1.0). Origin software was used for data analysis, and the results were expressed as mean ± standard deviation (mean ± SD).

## 5. Conclusions

In this study, the optimal amounts of CG, MG, and BT to add to the medium in order to maximize biomass and the production of phycoerythrin and polysaccharides in *P. purpureum* were determined using RSM forecasting models. According to the forecasting models, the biomass was primarily influenced by MG, while phycoerythrin concentration was mainly influenced by BT. Meanwhile, the concentration of polysaccharides was influenced by the interactive effects between CG and BT and between MG and BT. The maximum concentrations of biomass, phycoerythrin, and polysaccharides (5.97 g L^−1^, 102.95 mg L^−1^, and 1.42 g L^−1^) surpassed their predicted values and were reached when the microalgae were cultured under the optimal conditions indicated by the models. Hence, CG, MG, and BT can be considered as exogenous additives to greatly promote *P. purpureum* growth and the synthesis of phycocyanin and polysaccharides. As a next step, it is important to further increase the production of algal biomass, phycoerythrin, and polysaccharides by utilizing a larger-scale photobioreactor and optimizing light intensity during culture in the optimal medium identified in the present study.

## Figures and Tables

**Table 1:** Analysis of variance for the response-surface models.

Source	Modified Equations with Significant Terms	*p*-Value	R^2^	Adj.R^2^	SD	Lack of Fit	C.V.%
Biomass concentration	5.21 + 0.28A − 0.705B + 0.0925C − 0.1475AB + 0.4875AC − 0.0225BC − 0.1187A^2^ − 0.4137B^2^ − 1.01C^2^	0.0123	0.8893	0.7469	0.4449	0.3384	9.93
Phycoerythrin concentration	74.11 − 1.6A − 0.85B − 8.84C − 5.89AB − 1.42AC + 0.165BC + 1.99A^2^ − 4.96B^2^ − 24.28C^2^	0.0054	0.9145	0.8046	6.78	0.6649	11.07
Polysaccharide concentration	1.02 + 0.1588A + 0.1338B + 0.0525C + 0.0225AB + 0.11AC + 0.125BC − 0.0647A^2^ − 0.0748B^2^ − 0.1472C^2^	0.0054	0.9142	0.8039	0.0915	2.45	10.32

A, calcium gluconate; B, magnesium gluconate; C, polypeptides; R^2^, coefficient of determination; Adj.R^2^, adjusted R^2^; SD, standard deviation; CV, coefficient of variation.

**Table 2:** Experimental data and predicted values based on established models of biomass and phycoerythrin and polysaccharide concentrations.

Std	Run	Variables						Responses		
		CG		MG		BT		Biomass	Phycoerythrin Concentration	Polysaccharide Concentration
		(g L^−1^)		(g L^−1^)		(g L^−1^)		(g L^−1^)	(mg L^−1^)	(g L^−1^)
		Coded	Actural	Coded	Actural	Coded	Actural	Actural	Actural	Actural
1	5	−1	2	−1	8	0	2	5.77	84.23	1
2	13	1	6	−1	8	0	2	4.87	70.01	1.13
3	16	−1	2	1	16	0	2	4.02	56.78	0.76
4	8	1	6	1	16	0	2	4.36	36.91	0.64
5	6	−1	2	0	12	−1	0.5	5.28	69.73	0.52
6	17	1	6	0	12	−1	0.5	4.22	56.78	0.78
7	9	−1	2	0	12	1	3.5	4.91	44.05	1.06
8	7	1	6	0	12	1	3.5	3.78	60.77	1.29
9	3	0	4	−1	8	−1	0.5	3.32	43.79	0.64
10	14	0	4	1	16	−1	0.5	3.5	33.3	1.09
11	4	0	4	−1	8	1	3.5	5.31	65.09	1.03
12	10	0	4	1	16	1	3.5	5.33	72.4	1.02
13	2	0	4	0	12	0	2	6.08	72.02	0.91
14	15	0	4	0	12	0	2	3.25	52.51	0.71
15	12	0	4	0	12	0	2	4.77	78.83	0.93
16	11	0	4	0	12	0	2	3.57	82.06	0.81
17	1	0	4	0	12	0	2	3.86	62.7	0.76

CG, calcium gluconate; MG, magnesium gluconate; BT, polypeptide.

**Table 3:** Summary of algal growth, phycoerythrin production and polysaccharide production in *Porphyridium* sp. reported in the literature and in this study.

Number	Medium	Special Culture Conditions	Culturing Scale	Biomass Concentration or Cell Number	Polysaccharide Concentration (g L^−1^)	PB and PE Concentrations (mg L^−1^)	Refs
1	F/2	N: P ratio	250 mL flask	5.94 × 10^9^ cell L^−1^	0.23	NA	[46]
2	F/2-RSE	Light, temperature, and nitrogen	250 mL glass flask	3.4 g L^−1^	0.92	PB: 47.20 PE: 38.80	[29]
3	OMII	Consumption of N and P	30 L flat-plate photobioreactor	1.71 × 10^10^ cell L^−1^	0.73	NA	[47]
4	OMI	Light regime	15 L plate photobioreactor	1.38 × 10^10^ cell L^−1^	0.95	NA	[48]
5	ASW	Outdoor mass culture	72 L flat plate glass reactor	1.37 × 10^11^ cell L^−1^	1.32	NA	[49]
6	ASW	Different nitrogen concentrations	6 × 60 cm photobioreactor	5.53 g L^−1^	2.14	PB: 1010	[43]
7	Pm	Light, temperature and nitrogen	5 L photobioreactor	6.12 × 10^10^ cell L^−1^	4.10	NA	[44]
8	ASW	Optimization of light and sodium bicarbonate	3 L batch culture of photobioreactor	15.2 g L^−1^	4.5	PB 12.17 g/100 g	[45]
9	ASW	light intensities	BioIII fermenter	4.44 × 10^9^ cell L^−1^	4.63	NA	[21]
10	ASW	Addition of CG, MG, and BT	500 mL flask	5.97 g L^−1^	1.42	102.95	

**Table 4:** Independent variables (CG, MG and BT concentration) and the levels of each treatment used in the Box-Behnken design.

Parameters	Lable	Coded Levels and Concentrations		
(g L^−1^)		−1	0	+1
CG	A	2	4	6
MG	B	8	12	16
BT	C	0.5	2	3.5

CG, calcium gluconate; MG, magnesium gluconate; BT, polypeptides.

## Data Availability

All data generated or analyzed in the present study are available on reasonable request.

## Supplementary Materials

The following supporting information can be downloaded at https://www.mdpi.com/article/10.3390/md22030138/s1, Table S1: Analysis of variance for a quadratic model of biomass concentration; Table S2: Analysis of variance for a quadratic model of phycoerythrin concentration; Table S3: Analysis of variance for a quadratic model of polysaccharide concentration; Table S4: Effects of different exogenous substances on phycoerythrin and polysaccharide contents.
