# Supplementary material for: Maximizing Polysaccharides and Phycoerythrin in Porphyridium purpureum via the Addition of Exogenous Compounds: A Response-Surface-Methodology Approach
Source: Mar Drugs. 2024 Mar 21;22(3):138. doi: 10.3390/md22030138 (PMC10971926; doi:10.3390/md22030138)
Supplement: Supplementary file 1 [file marinedrugs-22-00138-s001.zip › marinedrugs-2915983-supplementary.pdf]

**Table S1** Analysis of variance for quadratic model for biomass concentration.

| Source | Sum of Squares | df | Mean Square | F-value | p-value | Remark |
|---|---|---|---|---|---|---|
| Model | 11.13 | 9 | 1.24 | 6.25 | 0.0123 | Significant |
| A-CG | 0.6272 | 1 | 0.6272 | 3.17 | 0.1183 | |
| B-MG | 3.98 | 1 | 3.98 | 20.09 | 0.0029 | Significant |
| C-BT | 0.0685 | 1 | 0.0685 | 0.3458 | 0.575 | |
| AB | 0.087 | 1 | 0.087 | 0.4397 | 0.5285 | |
| AC | 0.9506 | 1 | 0.9506 | 4.8 | 0.0645 | |
| BC | 0.002 | 1 | 0.002 | 0.0102 | 0.9223 | |
| $A^2$ | 0.0594 | 1 | 0.0594 | 0.3 | 0.6009 | |
| $B^2$ | 0.7208 | 1 | 0.7208 | 3.64 | 0.098 | |
| $C^2$ | 4.33 | 1 | 4.33 | 21.86 | 0.0023 | Significant |
| Residual | 1.39 | 7 | 0.1979 | | | |
| Lack of Fit | 0.7383 | 3 | 0.2461 | 1.52 | 0.3384 | Not significant |
| Pure Error | 0.6472 | 4 | 0.1618 | | | |
| Cor Total | 12.51 | 16 | | | | |

CG, calcium gluconate; MG, magnesium gluconate; BT, polypeptides.

**Table S2** Analysis of variance for quadratic model for phycoerythrin concentration.

| Source | Sum of Squares | df | Mean Square | F-value | p-value | Remark |
|---|---|---|---|---|---|---|
| Model | 3444.89 | 9 | 382.77 | 8.32 | 0.0054 | Significant |
| A-CG | 20.54 | 1 | 20.54 | 0.4466 | 0.5254 | |
| B-MG | 5.78 | 1 | 5.78 | 0.1257 | 0.7334 | |
| C-BT | 625.16 | 1 | 625.16 | 13.59 | 0.0078 | Significant |
| AB | 139 | 1 | 139 | 3.02 | 0.1257 | |
| AC | 8.01 | 1 | 8.01 | 0.1741 | 0.689 | |
| BC | 0.1089 | 1 | 0.1089 | 0.0024 | 0.9626 | |
| $A^2$ | 16.74 | 1 | 16.74 | 0.3639 | 0.5654 | |
| $B^2$ | 103.63 | 1 | 103.63 | 2.25 | 0.1771 | |
| $C^2$ | 2481.36 | 1 | 2481.36 | 53.94 | 0.0002 | Significant |
| Residual | 322 | 7 | 46 | | | |
| Lack of Fit | 96.21 | 3 | 32.07 | 0.5681 | 0.6649 | Not significant |
| Pure Error | 225.79 | 4 | 56.45 | | | |
| Cor Total | 3766.88 | 16 | | | | |

CG, calcium gluconate; MG, magnesium gluconate; BT, polypeptides.

**Table S3** Analysis of variance for quadratic model for polysaccharide concentration.

| Source | Sum of Squares | df | Mean Square | F-value | p-value | Remark |
|---|---|---|---|---|---|---|
| Model | 0.6249 | 9 | 0.0694 | 8.29 | 0.0054 | Significant |
| A-CG | 0.2016 | 1 | 0.2016 | 24.06 | 0.0017 | Significant |
| B-MG | 0.1431 | 1 | 0.1431 | 17.08 | 0.0044 | Significant |
| C-BT | 0.0221 | 1 | 0.0221 | 2.63 | 0.1488 | |
| AB | 0.002 | 1 | 0.002 | 0.2417 | 0.638 | |
| AC | 0.0484 | 1 | 0.0484 | 5.78 | 0.0472 | Significant |
| BC | 0.0625 | 1 | 0.0625 | 7.46 | 0.0293 | Significant |
| $A^2$ | 0.0177 | 1 | 0.0177 | 2.11 | 0.1899 | |
| $B^2$ | 0.0235 | 1 | 0.0235 | 2.81 | 0.1377 | |
| $C^2$ | 0.0913 | 1 | 0.0913 | 10.9 | 0.0131 | Significant |
| Residual | 0.0587 | 7 | 0.0084 | | | |
| Lack of Fit | 0.038 | 3 | 0.0127 | 2.45 | 0.2036 | Not significant |
| Pure Error | 0.0207 | 4 | 0.0052 | | | |
| Cor Total | 0.6836 | 16 | | | | |

CG, calcium gluconate; MG, magnesium gluconate; BT, polypeptides.

**Table S4** Effect of phycoerythrin and polysaccharide contents in different exogenous substances.

| Types | Concentration（g $L^{-1}$） | Phycoerythrin content（mg $g^{-1}$） | Polysaccharide content（g $g^{-1}$） |
|---|---|---|---|
| CG | 0 | 34.3±2.42 | 0.12±0.01 |
| | 2 | 43.6±1.25 | 0.19±0.008 |
| | 3 | 40.12±0.98 | 0.15±0.019 |
| | 4 | 30.67±2.7 | 0.16±0.013 |
| | 5 | 29.9±2.87 | 0.15±0.014 |
| | 6 | 30.17±2.97 | 0.15±0.008 |
| MG | 0 | 59.57±3.38 | 0.07±0.006 |
| | 10 | 39.52±1.14 | 0.2±0.1 |
| | 11 | 36.75±0.64 | 0.22±0.034 |
| | 12 | 54.67±2.99 | 0.17±0.008 |
| | 13 | 46.18±1.37 | 0.17±0.005 |
| | 14 | 43.85±5.68 | 0.22±0.015 |
| BT | 0 | 83.11±7.77 | 0.1±0.007 |
| | 0.5 | 90.32±6.12 | 0.09±0.005 |
| | 1 | 85.17±7.21 | 0.09±0.005 |
| | 1.5 | 75.09±5.23 | 0.1±0.003 |
| | 2 | 63.02±3.96 | 0.11±0.011 |
| | 2.5 | 71.19±4.5 | 0.12±0.004 |

CG, calcium gluconate; MG, magnesium gluconate; BT, polypeptides.
